# Sustainable Netting Materials for Marine and Agricultural Applications: A Perspective on Polymeric and Composite Developments

**DOI:** 10.3390/polym17111454

**Published:** 2025-05-23

**Authors:** Leonardo Pagnotta

**Affiliations:** Department of Mechanical, Energy and Management Engineering, University of Calabria, 87036 Rende, Italy; leonardo.pagnotta@unical.it

**Keywords:** polymeric nets, biodegradable composites, marine litter, agricultural netting, bio-based materials, multifunctional nets, netting life cycle

## Abstract

This review addresses the growing demand for sustainable alternatives to conventional synthetic nets used in marine and agricultural applications, which are often persistent, poorly degradable, and difficult to manage at end of life. It examines recent developments in biodegradable polymers—particularly polylactic acid (PLA), polyhydroxyalkanoates (PHAs), and poly(butylene adipate-co-terephthalate) (PBAT)—alongside reinforced blends and nanocomposites designed to improve mechanical performance and degradation behavior under real-world conditions. Strategies based on the regeneration of discarded nets, especially those made from polyamide 6 (PA6), are also considered for their potential to close material loops and reduce environmental leakage. A critical analysis of current testing protocols and regulatory frameworks is provided to assess their suitability for novel materials. In addition, this study highlights the emergence of multifunctional nets capable of providing environmental sensing or biological support, marking a transition toward adaptive and ecosystem-responsive designs. Finally, a survey of ongoing European and international projects illustrates scalable pathways for implementing biodegradable and recyclable netting systems, integrating material innovation with circular economy strategies. These findings emphasize the need for harmonized standards, targeted environmental testing, and cross-sectoral collaboration to enable the large-scale adoption of sustainable net technologies.

## 1. Introduction

Nets are engineered mesh systems with a remarkably broad range of applications, spanning aquatic environments—such as fishing, aquaculture, and installations in marine and freshwater settings, including containment barriers and monitoring structures—to terrestrial uses in agriculture, particularly for crop protection, wildlife exclusion, and fencing. Their use also extends to civil and construction sectors, where they serve as structural reinforcements, fall protection systems, and debris containment solutions. In addition, nets are employed across various other fields, including logistics, sports, and urban infrastructure, reflecting their versatility as lightweight, permeable, and adaptable systems. Given such functional diversity, the material used for these nets largely determines their performance, durability, and environmental impact. The growing focus on sustainability has led to a critical reassessment of the materials employed, steering research toward lighter, more efficient, biodegradable solutions or those derived from recycling processes.

Polymeric and composite nets, due to their lightness, flexibility, chemical resistance, and ease of processing, currently represent the predominant category in high-consumption sectors. However, applications involving natural fibers (e.g., sisal, hemp) and metals still persist in structural, artisanal, or heavy-duty protection contexts. Unlike natural or metallic alternatives, polymeric and composite systems can be designed to be modular and multifunctional, allowing the integrated optimization of functional properties.

Within this broad spectrum of uses, the present work focuses on two particularly significant and strategic sectors: fisheries and agricultural applications. While marine fisheries and offshore aquaculture are exposed to harsh environmental conditions—such as high salinity, UV radiation, and dynamic mechanical loads—freshwater systems, including river and lake fishing or inland aquaculture installations, are generally subject to milder conditions. Nonetheless, these too require adequate mechanical stability and resistance to biofouling, sediment accumulation, or biological degradation. In marine contexts, accidental net losses remain one of the main sources of plastic pollution in oceans (ghost fishing), with serious ecological consequences [1]. Agricultural nets, widely employed for crop protection or microclimate regulation, are subject to sunlight and weathering, leading over time to material degradation and potential waste generation [2,3]. In both fisheries and agriculture, there is a clear need for materials designed not only for performance but also with their end of life in mind.

Historically, the use of net-like materials based on natural fibers dates back to ancient times [4]. The introduction of synthetic fibers such as nylon, polyethylene, and polypropylene marked a technical turning point in the second half of the twentieth century, significantly improving mechanical performance and durability [5]. In recent years, attention has gradually shifted toward biodegradable materials and recycled composites, with examples ranging from compostable polymers (PLA, PHA, PBS, PBAT) [6,7] to the reuse of discarded nets as reinforcement in construction materials [8].

The main objectives of this review are to provide a comprehensive and up-to-date overview of polymeric and composite materials used in netting systems for marine and agricultural applications, and to evaluate their mechanical performance, environmental behavior, and suitability for sustainable development. The review classifies the main types of net structures and materials, analyzes physical and mechanical properties relevant to field performance, and compares traditional polymers with emerging solutions such as biodegradable blends, nanocomposites, and recycled materials. It also examines regulatory requirements and testing approaches, offering a framework to support the design of next-generation sustainable netting technologies.

### Methodological Framing

This review adopts a narrative and comparative approach to examine polymeric and composite materials used in marine and agricultural netting systems. It integrates mechanical, environmental, and regulatory perspectives with the aim of identifying recent trends and knowledge gaps.

The analysis covers the period from 2010 to 2024 and includes peer-reviewed articles retrieved from Scopus, Web of Science, and Google Scholar using keywords such as *biodegradable polymers*, *fishing nets*, *agricultural nets*, *polymer degradation*, and *recycled plastics*.

The inclusion criteria focused on relevance to netting applications, the presence of experimental or technical data, and thematic alignment with sustainability and material innovation.

Notably, more than half of the cited sources were published from 2022 onward, confirming the topicality and up-to-date nature of this review. In addition to the scientific literature, the review also considers institutional publications and publicly available data from national and international initiatives, offering further insights into ongoing developments and real-world applications.

## 2. Types, Applications, and Materials for Technical Nets

**Figure 1:**
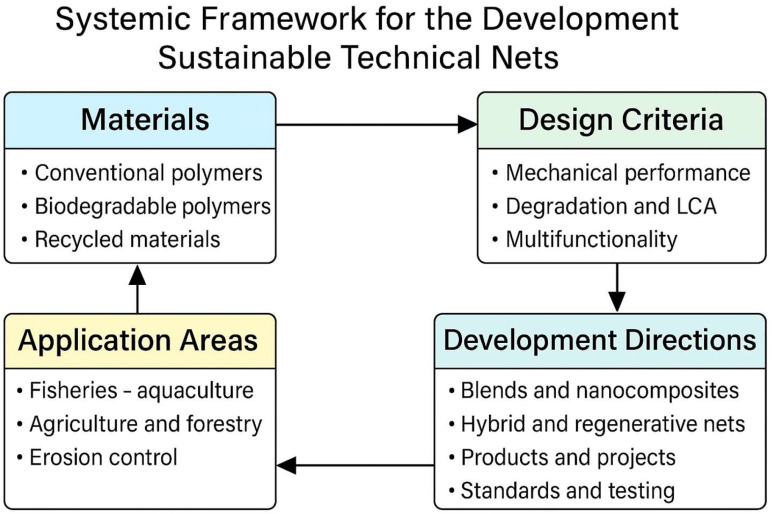
Systemic framework for the development of sustainable technical nets. The scheme outlines the interconnections between materials (conventional, biodegradable, recycled), design criteria (mechanical performance, multifunctionality, degradation and LCA), directions of innovation (composites, hybrid systems, products, standards), and key application sectors (fisheries, agriculture, erosion control).

The development of technical nets for marine and agricultural use requires a systemic approach, encompassing materials, design criteria, and application-driven innovation. Figure 1 summarizes the conceptual framework guiding this review, highlighting the interconnected factors that influence the evolution of sustainable netting systems.

The historical evolution of materials and technologies used in net manufacturing provides an essential interpretive key to understanding current structural features, application contexts, and future development prospects. Before analyzing the various types and most widely used materials today, a brief review is offered on the progression from ancient plant fiber nets to today’s innovative composites.

Although technical nets are employed in both marine and freshwater environments, their use in inland waters has progressively declined due to environmental and regulatory factors [9]. In the following, focus is placed on marine and coastal applications, where technical nets remain predominant.

The use of nets as functional systems for containment, protection, or capture is documented as far back as the Mesolithic era, as evidenced by the Antrea net (circa 8300 BC), made from willow fibers [4]. For centuries, net production relied exclusively on natural materials such as flax, hemp, nettle, and rush, selected for local availability and mechanical properties and treated with substances like wax or pitch to improve durability. With 19th-century mechanization, production techniques became more standardized, but the decisive turning point came after World War II with the introduction of synthetic polymers. Materials such as polyamide, polyethylene, and polypropylene gradually replaced natural fibers due to their superior toughness, environmental stability, and durability [3,10]. This transition expanded the use of nets into areas such as agriculture, aquaculture, environmental protection, and civil engineering. In specific applications such as fishing pots or weighted cages, metallic structural elements (e.g., steel or copper wire) are still used in combination with the main net [10]. Additionally, synthetic fibers recovered from discarded nets are now reused as reinforcement in cementitious and gypsum-based mortars, contributing to the development of sustainable composite materials [8]. In recent years, growing environmental awareness has led to the development of new bio-based or biodegradable materials, including PLA, PBS, PBAT, and PHA, sometimes in advanced configurations such as nanocomposites or bioinspired fibers [7,8,11]. The evolution of materials thus reflects a progressive transformation of nets from handcrafted natural fiber products to complex technical systems where mechanical performance, durability, and sustainability are synergistically combined according to application needs.

Marine and agricultural nets now feature a remarkable variety of shapes, structures, and materials, tailored to specific operational environments, mechanical functions, and life cycle requirements. A systematic classification can be carried out according to three main criteria: structural configuration, intended use, and material type.

### 2.1. Classification by Structural Configuration

The first distinction concerns the mesh structure, which may consist of continuous or discontinuous filaments, with or without knots (knotless), either twisted or woven, and arranged in braids, cords, or grids. Knotted nets (common in many artisanal and industrial fishing applications) offer high dimensional stability, while knotless nets are lighter and deform more uniformly under load [5].

The structure of the filaments may vary, giving rise to nets composed of the following:

- Monofilaments: continuous single fibers that provide strength but low elasticity.
- Multifilaments: aggregated or textured fibers, more flexible and deformable.
- Twisted or braided cords: used where higher robustness or cut resistance is required.
- Woven or knitted nets: commonly used in agriculture and covering systems.

Some fishing net configurations, especially those used for selective capture, employ combinations of varied geometries and multilayer materials to optimize efficiency and reduce bycatch [9]. Recently, biomimetic configurations have also been introduced, such as core–shell fibers inspired by mussel byssus, which enhance flexibility and mechanical response under dynamic loads [11].

The structural configurations of technical nets vary depending on the application context (marine or agricultural), the type of load, the expected service life, and environmental compatibility. Table 1A,B present a functional classification that distinguishes between well-established solutions and less common or emerging configurations, highlighting for each the typical composition, mechanical characteristics, and predominant application sectors.

Table 1A,B exhibit a functional classification of the main structural configurations of technical nets. Table 1A includes the most widespread configurations in marine and/or agricultural contexts, while Table 1B presents emerging or specialized types, with experimental or niche applications. For each configuration, the commonly used materials, relevant technical characteristics, and key bibliographic references are provided.

### 2.2. Classification by Intended Use

Depending on the context of use, technical nets exhibit widely varying structural configurations and physical properties.

#### 2.2.1. Nets for Marine Applications

These include fishing nets (trawl, gill, and purse seines), aquaculture cages, and containment nets that must withstand dynamic loads, prolonged immersion in saltwater, biofouling, and UV radiation. Durability is a key requirement, but there is a growing demand for controlled biodegradability to reduce environmental impact in the event of accidental loss [1,6]. Over the years, several products have been developed to address the problem of lost fishing gear. For example, Bioline™ was an early biodegradable fishing line, designed to retain its mechanical strength for about 10–12 months before beginning degradation [15]. Although Bioline™ is no longer commercially available, it demonstrated potential as environmentally responsible fishing gear.

More recently, TUF-LINE Biodegradable Monofilament has been introduced, offering a plant-based fishing line that maintains its strength for one year before biodegrading completely in natural environments [16]. In parallel, Biodolomer^®^Ocean Nets have been developed as fully marine-biodegradable fishing nets, providing a sustainable alternative to conventional polyethylene nets [17].

#### 2.2.2. Nets for Agricultural Applications

In agriculture, nets are used for shading, hail protection, insect exclusion, windbreaks, mulching, and the containment of crop residues. The required properties vary according to function: selective optical transparency for shading nets, dimensional stability for hail nets, and air permeability and tensile strength for insect nets. Here, too, the need for biodegradability, low weight, and ease of disposal is increasingly important [2,3,10]. Recent research, such as that of Maraveas [18], has proposed an integrated approach based on life cycle assessment (LCA), aimed at comparing the overall environmental impact of bio-based materials with conventional ones, considering the entire life cycle.

Within this context, recent studies have explored surface treatments applied to biodegradable polymers intended for agricultural nets. Knoch et al. [19] demonstrated that PLA nets produced using dual solvent diffusion (DDD) and plasma-induced chemical vapor deposition (PICVD) exhibit enhanced hydrophobicity and reduced moisture absorption. These treatments contribute to improved stability under variable environmental conditions, making them suitable for seasonal agricultural use or for applications requiring controlled degradation.

Nets are also used in civil and environmental engineering for soil stabilization, rockfall protection, hydraulic works, and the containment of bulk materials. In many of these applications, polymeric or metallic nets are employed, often in combination with geotextiles or composite materials, ensuring structural lightness, installation flexibility, and good performance in harsh environments.

**Figure 2:**
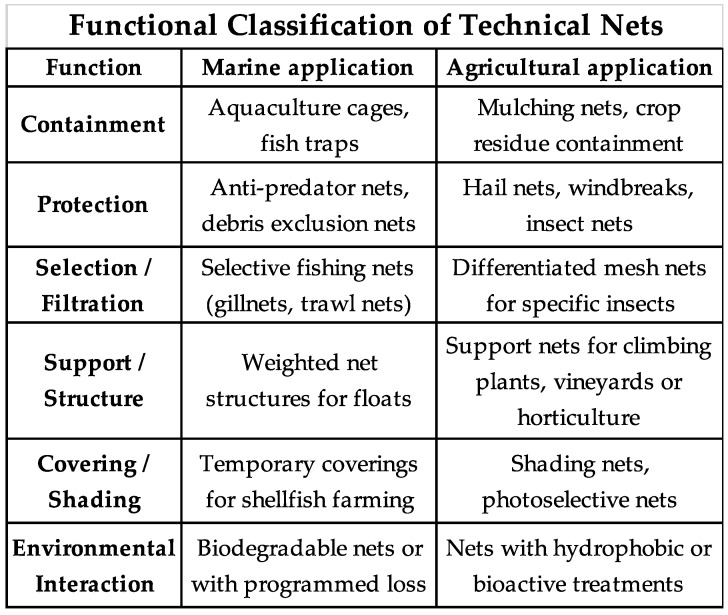
Functional classification of technical nets in marine and agricultural applications. The table cross-references the main operational functions (containment, protection, selection, support, coverage, environmental interaction) with specific examples related to the two application contexts.

To facilitate an overview of the primary functions of nets in marine and agricultural contexts, Figure 2 presents a cross-functional classification that associates different applications with their corresponding operational roles. This diagram highlights parallels across sectors and supports the selection of design solutions based on the specific context of use.

### 2.3. Classification by Material

The third classification criterion concerns material composition, which affects mechanical properties, durability, environmental compatibility, and processes of manufacturing and recycling.

This subsection provides a technical overview of the main materials used in net production, including conventional polymers, bio-based alternatives, recycled composites, and metallic components. Emphasis is placed on how material choice influences net behavior under operational conditions, long-term performance, and environmental impact.

#### 2.3.1. Conventional Materials

The earliest nets were made from natural fibers such as flax, hemp, cotton, and sisal, now largely replaced due to their poor durability in humid environments and maintenance difficulties [4]. Today, the most widely used materials include the following:

- Polyamides (PA-6, PA-6.6): High strength, elasticity, good chemical stability; widely used in fishing and some agricultural nets. Studies by Moe et al. (2007) [12] showed that the tensile stiffness of PA nets increased significantly after antifouling treatments, with an average stiffness of about 81 MPa for untreated nets and approximately 131 MPa for treated ones.
- Polyethylene (HDPE, LDPE): good chemical resistance, low cost, low density, but relatively low tensile strength.
- Polypropylene (PP): lighter than PE but less resistant to UV radiation.
- Polyethylene terephthalate (PET): excellent mechanical and thermal resistance, also used in next-generation nets [5].

#### 2.3.2. Innovative and Sustainable Materials

In recent years, there has been growing interest in biodegradable or recycled materials, including the following:

- PLA (Polylactic Acid): biodegradable under industrial composting, with limited mechanical performance in marine environments [6].
- PHA and PHB: biodegradable in seawater but still limited in large-scale use due to cost.
- PBAT (Polybutylene Adipate-co-Terephthalate): flexible, biodegradable, also used in nanostructured composite fibers [7].
- PBS (Polybutylene Succinate): good initial strength and controlled degradability; used in biodegradable fishing nets [1].
- PBAT/PBS Core–Shell Fibers: inspired by mussel byssus, designed to ensure strength and drapability [11].
- PBAT/CNC Nanocomposites: reinforced with cellulose nanocrystals, achieving performance comparable to nylon [11].
- Recycled Composites: regenerated nylon from discarded nets, reused in construction materials or low-impact nets [8].

Recent studies show that synthetic nets undergo progressive degradation in real marine environments. Liu et al. [20] documented an average loss of 52% in tensile strength in aquaculture nets after three years of service, highlighting the importance of long-term durability assessments in material selection.

In addition to fully biodegradable and recycled materials, recent studies have investigated semi-biodegradable blends, in which biodegradable matrices such as PLA or PBAT are combined with conventional polymers like ABS or EVA. These systems, though not fully compostable, are engineered to enhance impact resistance, ductility, and processing stability, providing a compromise between biodegradability and functional reliability.

For instance, Santos Filho et al. (2025) [21] developed PLA/ABS blends compatibilized with epoxy-functionalized SAN (styrene-acrylonitrile) copolymers. Their results showed that a PLA/ABS (70/30) formulation, modified with 10 phr SAN–epoxy, achieved a 200% increase in impact strength and improved phase dispersion compared to neat PLA. These enhancements were attributed to better interfacial adhesion and finer morphology observed via SEM analysis.

Similarly, Chen et al. (2025) [22] investigated ternary PLA/PBAT/EVA systems toughened using a multifunctional epoxy chain extender. Their study demonstrated that the optimal formulation reached an impact strength of 51.99 kJ/m^2^, more than 22 times higher than PLA, and an elongation at break of 482%, while maintaining tensile strength around 43.5 MPa. The compatibilized blend also showed improved thermal and morphological stability.

Although these semi-biodegradable systems are not suitable for fully degradable monofilaments, they represent a promising option for auxiliary or structural components in netting systems—such as protective reinforcements, connectors, or edge zones—where a balance between durability and partial environmental compatibility is required.

#### 2.3.3. Metallic Materials and Hybrid Structures

Metallic nets, although less common in flexible mesh fabrics, are employed in specific contexts requiring increased structural stiffness and long-term resistance in harsh environments. In marine applications, steel or copper wire elements are utilized in traps, in aquaculture cages, and as ballast components in purse seine nets, providing geometric stability and abrasion resistance (PEsca Senza PLAstica, 2022) [23].

In civil and environmental engineering, coated or galvanized steel nets are widely used for slope stabilization, rockfall protection, and the containment of loose materials, often combined with geotextiles or composite layers.

The interaction between metallic support structures and flexible nets induces localized mechanical stresses, promoting wear at contact points. Despite their high mechanical resistance, metallic nets are heavier, less manageable, and susceptible to corrosion, and are therefore primarily reserved for structural reinforcements rather than for full-net fabrication.

The selection of material and structural configuration depends strictly on the intended application and must integrate performance requirements, operational conditions, durability, and end-of-life considerations. These aspects influence physical and mechanical properties, design strategies, and the development of advanced and sustainable netting materials, as discussed in subsequent sections.

## 3. Properties and In-Service Performance

Nets used in marine and agricultural applications are subject to complex stresses, harsh environments, and repeated use cycles, all of which influence durability, functionality, and end-of-life management. The required properties of mesh materials go beyond mechanical strength and increasingly include flexibility, dimensional stability, environmental resistance, and controlled biodegradability. This section analyzes the main characteristics required, focusing on polymeric and composite materials relevant to marine and agricultural applications.

### 3.1. Mechanical Properties

The most relevant mechanical properties are tensile strength, elastic modulus, tear resistance, and, in many cases, dynamic resilience (the ability to absorb impact loads without failure).

Table 2 organizes the main mechanical properties of polymers used or proposed for fishing and agricultural netting, distinguishing between conventional fossil-based materials and biodegradable or recycled alternatives. This comparative framework allows for an evaluation of key parameters—tensile strength, stiffness, and elongation—across different material classes and formulations. Fossil-based polymers, such as PA6, HDPE, and PP, offer excellent mechanical reliability, making them suitable for demanding and long-term applications. In contrast, biodegradable polymers and their composites generally display lower mechanical performance, but show promise in contexts where short-term use, reduced environmental impact, or ease of biodegradation is prioritized. Reinforced formulations, such as PLA/flax or PA6/biochar, demonstrate measurable improvements in stiffness and strength, suggesting potential for targeted applications—particularly in seasonal or degradable netting systems.

Although the mechanical performance of fossil-based polymers remains unmatched for demanding applications such as long-term fishing or agricultural netting, Table 2 shows that biodegradable materials and their composites offer increasingly viable alternatives—especially for temporary or seasonal systems. While neat biopolymers like PLA and PBS exhibit lower strength and limited ductility, the incorporation of reinforcements (e.g., flax fibers or biochar) significantly enhances tensile and flexural properties. These improvements, however, should be evaluated in light of application-specific requirements, including exposure time, mechanical loading, and environmental degradation. Composite formulations, especially those based on recycled or bio-based matrices, offer an opportunity to tailor material performance and environmental impact, without necessarily aiming to replace high-performance PA or polyolefin nets in all use cases.

Fishing nets, for instance, must endure high cyclic loads during capture and retrieval, while agricultural nets must retain integrity after extreme weather events such as hail or strong winds.

PA (polyamide) nets offer good static and dynamic performance, with high toughness and elastic recovery [35]. HDPE and PP materials provide higher stiffness but less deformability and reduced aging resistance. The inclusion of reinforcing fillers, as in PBAT/CNC nanocomposites, enables a favorable balance of strength and ductility, as demonstrated by recent studies on biodegradable fibers [7].

Experimental and numerical analyses of agricultural nets show that fiber orientation and knot geometry significantly influence internal stress distribution and tensile behavior [36].

Specifically, Castellano et al. [13] showed that porosity, filament geometry, and knot type affect the aerodynamic drag coefficient in HDPE agricultural nets, enabling the formulation of useful correlations for designing passive ventilation and microclimate protection systems.

Halim [37] further demonstrated that the dimensional stability of certain polyolefin nets is retained even after long-term exposure, making them suitable for seasonal or semi-permanent use.

Several studies, including Castellano et al. [10], Sharif et al. [35], and Briassoulis [2], have highlighted the influence of structural configuration and filament type on the mechanical performance of agricultural nets.

Leno weave monofilament nets are generally more rigid and dimensionally stable, whereas multifilament Raschel nets exhibit greater adaptability and deformation capacity, with elongation up to about 50% before failure, depending on the material properties and structural geometry.

Sharif et al. (2021) [35] conducted a systematic comparative analysis of the effects of material, yarn diameter, and mesh size on fishing net strength. The results showed that PA nets provide the best tensile and elongation performance, followed by PE and PP. A larger yarn diameter increases breaking strength, while wider meshes are better suited for dynamic loads due to higher elastic energy dissipation. These observations provide useful design guidelines for selecting material and geometry based on the specific application.

From a reuse perspective, Singh et al. [38] evaluated regenerated PA nets as reinforcement in hybrid composites with glass fibers and polyester resins. The results showed increased bending and tensile strength compared to the pure matrix, suggesting the potential to extend the useful life of nets in new technical applications.

Similarly, Spadea et al. [39] explored the use of polyamide fibers from discarded fishing nets as reinforcement in cementitious mortars. Experimental results showed a 35% increase in first-crack strength and a 13-fold increase in toughness compared to the unreinforced matrix, indicating the reuse potential of such materials in construction and infrastructure.

Experiments in recent projects, although peripheral to mainstream developments, have validated the use of biodegradable fibers like Lyocell and PLA in marine environments. These materials demonstrated modest initial strength but adequate performance for short-term use, maintaining mechanical properties for several weeks [23].

### 3.2. Environmental Behavior

The operating environment is a crucial factor. Fishing and aquaculture nets operate in saline environments, sometimes at variable temperatures, and are subject to biofouling, exposure to UV radiation, hydrolysis, and continuous mechanical abrasion. Agricultural nets, although not immersed, are still exposed to atmospheric agents (rain, wind, solar radiation), daily thermal cycles, and photoinduced oxidation phenomena.

The physical–chemical changes induced by the environment determine the loss of tenacity, yellowing, surface embrittlement, and reductions in tensile strength.

A significant example is provided by Thomas [40], who studied the behavior of polyamide 6 yarns exposed to solar radiation for 180 days. The results show a reduction in breaking load greater than 50% and a progressive decrease in elongation at break, highlighting the vulnerability of PA6 to environmental photodegradation processes.

Some materials such as PET exhibit good resistance to photo-oxidation and moisture but remain poorly degradable, while others such as PLA or PHA, although biodegradable under industrial conditions, are sensitive to hydrolysis and show variable degradation behavior in uncontrolled natural environments [1,6].

Castellano et al. [41] discussed the influence of polymer type, additives, and net structure on the long-term durability of HDPE and PP agricultural nets. Their analysis highlights how exposure to UV radiation can significantly affect the mechanical strength and appearance of these materials, depending on the presence and effectiveness of stabilizing additives.

Exposure tests at sea confirm that Lyocell can completely degrade in a few months, while PLA tends to resist for longer periods without evident signs of degradation, suggesting its preferential use in protected contexts or with programmed decomposition [23].

More recent studies [19] have introduced surface treatments of PLA, including double solvent diffusion (DDD) and film deposition by PICVD technology, with the aim of improving the water repellency of the material and reducing its moisture absorption. These modifications, while not affecting the biodegradability of the material, allow for the controlled prolongation of the dimensional stability of PLA in outdoor agricultural environments.

As an alternative to surface treatments, encouraging results have also been obtained through the modification of polymeric blends. Rossi et al. [26] have shown that the addition of biochar from lignocellulosic biomass to PA6 recycled from fishing nets allows for an increase in the elastic modulus from 2.6 to 4.5 GPa and a reduction in water absorption from 3.6% to 1.8%. This solution allows for improvements in durability in humid environments and reductions in the density and costs of the material, maintaining good mechanical properties for lightweight applications.

These surface modification interventions are particularly effective in protected agriculture, where moisture control and the dimensional stability of nets are essential. The resulting hydrophobic properties, as demonstrated for PLA and for biochar-modified PA6 recycled from fishing nets [19,26], help to optimize the microclimate and extend the life of seasonal covers.

### 3.3. End-of-Life Behavior and Biodegradability

One of the central aspects of contemporary net design is the integration of end-of-life behavior into material selection criteria. Non-recovered nets are a source of environmental pollution, both in agricultural systems and in marine ecosystems. Non-degradable polymers, such as PA and PE, can persist for decades in ecosystems, fragmenting into microplastics without fully disintegrating.

Recent regulations, such as the ASTM D6691 on marine degradation, establish rigorous criteria to qualify materials as biodegradable [6]. PHA, for example, has shown significant biodegradation in marine environments within 180 days, while PLA tends to degrade only under industrial composting conditions. PBAT fibers offer greater application flexibility, being degradable in both aerobic and anaerobic environments [7].

Recent experiences have verified the possibility of using biodegradable materials also for real fishing nets. The PEsca Senza PLAstica project (2022) developed prototypes of pots and gillnets using PLA and Lyocell yarns, testing them at sea. The tests showed sufficient resistance for seasonal use and progressive degradation within a few months.

However, not all PLA formulations are suitable for the marine environment. Greene [6] observed that under standardized ASTM conditions, some PLA-based products do not reach significant levels of biodegradation, while PHA formulations showed greater effectiveness. This underlines the importance of specific tests depending on the material, geometry, and operating environment.

Among the most suitable materials for fishing nets and agriculture are also PBS, already used in commercial solutions with a predetermined duration, and PBAT/CNC composites, capable of offering mechanical resistance comparable to that of technical polymers. In parallel, the recovery and regeneration of materials such as nylon (PA6) from disused fishing nets represent an effective strategy to reduce waste and promote the circular economy. Although not biodegradable, PA6 can be successfully reintegrated into new products, thereby reducing the environmental persistence of ghost gear [8].

**Figure 3:**
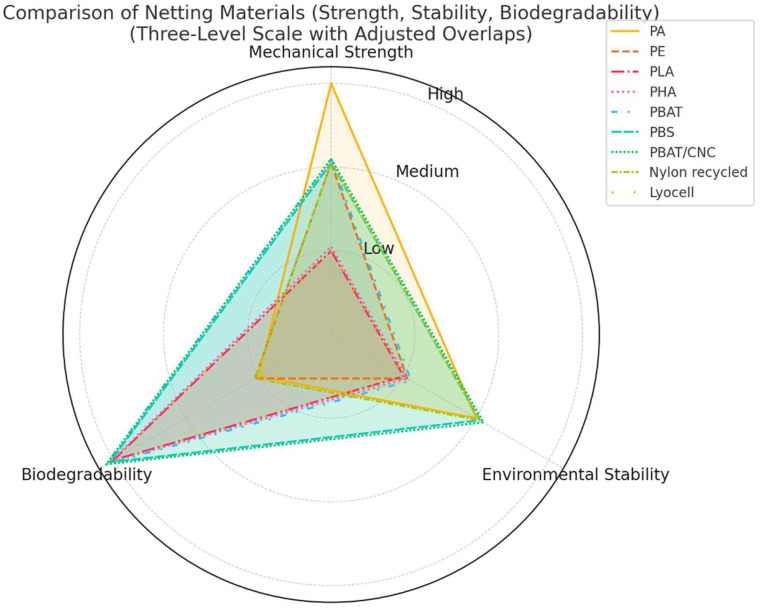
Radar comparison of main netting materials: This radar chart compares key netting materials in terms of mechanical strength, environmental stability, and biodegradability, using a simplified three-level qualitative scale (low, medium, high) with adjusted overlaps. The materials include conventional polymers (PA, PE), bio-based alternatives (PLA, PHA, PBS, PBAT, PBAT/CNC), recycled polymers (recycled nylon), and natural fibers (Lyocell). Conventional materials exhibit excellent strength and stability but limited biodegradability, while bioplastics and natural materials demonstrate more sustainable profiles with varying trade-offs in performance. Values are qualitatively estimated based on the literature and experimental data presented in the text.

Figure 3 visually summarizes the comparison between the main materials used or proposed, considering three fundamental parameters: mechanical resistance, environmental stability, and biodegradability. The radar representation allows one to immediately grasp the strengths and limitations of each solution, to support design selection.

## 4. Design Criteria and Regulatory Framework

The design of polymeric or composite nets for marine and agricultural applications goes beyond the selection of material and geometry. It requires an integrated approach that considers mechanical performance, durability, environmental impact, and regulatory compliance. Alongside traditional empirical methods, numerical and analytical modeling tools—supported by experimental data from real-world conditions—are becoming increasingly adopted to validate and optimize design solutions.

This section examines the main design criteria and the current regulatory framework, with reference to international standards and emerging best practices.

**Figure 4:**
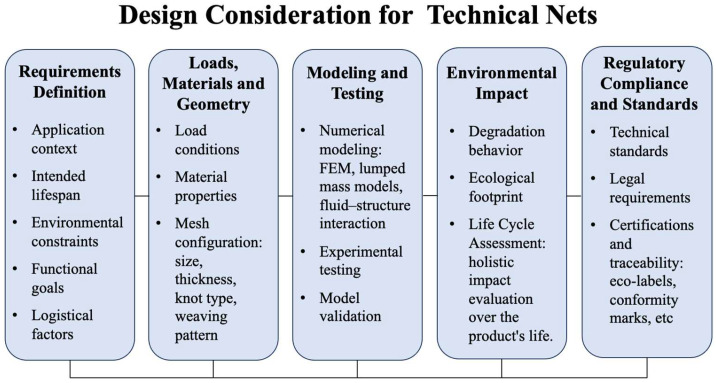
General design workflow for technical nets.

A general overview of the design methodology is presented in Figure 4, which outlines the five essential domains of sustainable net development: requirement definition, evaluation of loads and mesh configuration, modeling and testing, environmental impact assessment, and compliance with regulatory standards. The workflow emphasizes the iterative nature of simulation and field validation to ensure realistic, context-specific solutions.

This diagram outlines the five foundational domains guiding the design of polymeric and composite nets for marine and agricultural use: requirement definition, loads, materials, and geometry, modeling and testing, environmental impact, and regulatory compliance. Each block highlights key evaluation tasks necessary for optimizing performance, durability, and sustainability. Rather than following a strictly sequential process, these domains interact dynamically. In particular, feedback between modeling and experimental validation is essential for aligning technical predictions with real-world conditions and ensuring a robust, context-adapted design strategy.

### 4.1. Design Approach: Performance, Environment, and Life Cycle

#### 4.1.1. Fishing Nets: Simulation, Materials, and Controlled Degradation

The design of fishing nets involves balancing structural performance, environmental interaction, and end-of-life behavior. The main objectives concern maximizing catch efficiency (selectivity), resistance to complex loads (dynamic response), stability in marine environments, and, in more recent developments, controlled degradation to reduce environmental impact in case of gear loss.

The concept of selectivity, referring to a net’s ability to retain target species based on mesh size and deformability, was first introduced by Baranov [42], establishing the basis for rational net design. Mesh geometry directly affects the following:

- Catch selectivity;
- The hydrodynamic behavior of the panel (drag, vibration, resistance);
- Specific weight and net deformability;
- Material consumption and ease of retrieval.

Early empirical models, particularly those by Prado and Dremière for the FAO [43], offered preliminary geometric criteria, yet their validity needs reassessment for modern materials and operational contexts.

Analytical studies by Kawakami (1959, 1964) and Aarsnes et al. (1990) provided formulae for hydrodynamic forces on nets under steady currents, as summarized by Tsukrov et al. [44]. While useful for preliminary assessments, these models could not capture the dynamic deformation and interaction effects characteristic of flexible net panels.

The evolution of numerical tools introduced finite element modeling (FEM) into net design:

- Morison’s equation [45], originally formulated for estimating forces on rigid cylinders, was adapted to net structures, providing a theoretical foundation for many FEM approaches.
- Tsukrov et al. [44] developed an FEM model extending Morison’s framework to flexible nets, considering drag, added mass, buoyancy, elasticity, and self-weight. Their simulations accurately predicted deformations, inclination changes, and stress distribution under marine environmental loads, offering valuable guidance for choosing initial tensioning and mesh configurations.
- Cifuentes and Kim [46] introduced an equivalent-net Morison-force model, analyzing the effects of the solidity ratio (Sn) and flow velocity. Their refined drag coefficient (Cd) formulation, validated against Raschel net experiments, highlighted the importance of shielding effects, especially at high solidity.
- Casanova et al. [47] developed an FEM model in ABAQUS using Timoshenko beam elements to simulate the static behavior of PET nets under distributed loads. Experimental validation confirmed the model’s reliability even for rigid configurations, promoting its use for low-impact technical nets.

Parallel to these developments, simplified modeling approaches were explored to reduce computational costs:

More advanced developments have combined fluid and structural analyses to better model net deformation under realistic marine conditions:

- Moe-Føre [48] applied a lumped mass model to simulate aquaculture net deformations under hydrodynamic loading, using discrete mass–spring networks and empirical hydrodynamic coefficients. Although effective for capturing global behavior, this approach lacked full fluid–structure interaction coupling.
- Chen [49] advanced lumped mass modeling by integrating porous media theory, updating hydrodynamic loads dynamically based on net deformation. His model significantly improved the prediction of drag forces and structural responses in flexible nets subjected to currents and waves.
- Building upon Chen’s work, Zhang [50] introduced dynamic permeability updating and validated the model against both experimental flume tank tests and field measurements. His results demonstrated enhanced accuracy in predicting net deformation, drag reduction phenomena, and flow-induced effects, paving the way for more realistic simulations of net structures under operational conditions.

In parallel, Khawaja [51] proposed simplified numerical analyses combined with mechanical characterization of nets. While useful for initial assessments, these models did not achieve the predictive capabilities of FEM or lumped mass–porous models and remain limited to basic load–deformation evaluations.

Experimental studies continued to highlight the importance of construction details:

- Tang et al. [52] demonstrated, through flume tank experiments, that knot presence, yarn twist, and mesh orientation could influence hydrodynamic drag coefficients by up to 25%, confirming that detailed construction features significantly affect operational performance.

Despite the advances in structural and hydrodynamic modeling, modern net design must also incorporate environmental sustainability considerations. Post-use effects, such as ghost fishing and material persistence in the marine environment, have become critical drivers in the development of new gear solutions. In particular, the design of biodegradable nets aims to balance functional durability with programmed degradation, reducing long-term ecological impact in case of loss.

The *PEsca Senza PLAstica* project [23] monitored PLA and Lyocell nets in real environments, observing >30% strength loss in four months. Kim [1] and Kim et al. [53] documented the functional degradation of PBS nets within 90 days, while Gilman [54] analyzed the programmed degradation of PBAT and PBS pelagic nets over 24 months.

A recent review by Fan [9] summarized current approaches for simulating biodegradable nets in marine environments, confirming the need to combine numerical data, tank tests, and field trials to validate mechanical and environmental performance.

Numerous experimental applications have already shown successful implementation of the described design criteria in operational contexts. Selected case studies are summarized below to illustrate the complete design process, from objective definition to material selection, experimental validation, and assessment of environmental performance.

One of the most extensively documented cases is the design of PLA trammel nets for mullet fishing by Yu et al. [55]. The goal was to determine whether PLA could replace conventional PA while maintaining comparable functionality, with programmed degradation to reduce ghost fishing risks. The net was designed with the same configuration as standard PA nets but made from PLA. Sea trials in the Yellow Sea monitored catch efficiency, structural integrity, and degradation over time. The results showed that PLA nets retained comparable performance during the initial weeks, followed by progressive strength loss that reduced functionality and environmental risk—proving suitable for seasonal, low-intensity use.

A second example comes from the *PEsca Senza PLAstica* project [23], which developed and tested biodegradable mussel socks made from PLA and Lyocell. The objective was to design tubular nets capable of supporting mollusks during farming and degrading within a timeframe compatible with production cycles. Mesh size and elasticity were optimized to retain mussels, using environmentally compatible materials. Field trials assessed strength, biofouling, and end-of-life behavior. The results showed that PLA retained structural strength for about three months with subsequent degradation, while Lyocell degraded more rapidly. Both materials proved promising for seasonal or single-use devices.

A third case study, described by Kim et al. [1], involved pelagic PBS nets designed to maintain initial catch performance and disintegrate after a set period. The nets were designed similarly to PA nets, optimizing yarn diameter and mesh size for required strength during the first weeks. Mechanical and environmental tests were conducted in tanks and open water, simulating degradation under controlled conditions. The nets remained fully functional for two months, and then progressively lost structural integrity within 90 days—achieving short-term operational effectiveness while reducing long-term ghost fishing risk.

Emerging regulations, such as the EU Single-Use Plastics (SUP) Directive and new EMFAF (European Maritime, Fisheries and Aquaculture Fund) criteria, promote the development of biodegradable, taggable, and traceable fishing gear to reduce ghost fishing and facilitate gear recovery. In this framework, net design must consider not only in-service performance but also post-loss environmental behavior, assessing parameters such as fragmentation, buoyancy, biofouling, and actual degradation in marine environments.

These considerations highlight the need to integrate environmental, performance, and regulatory requirements from the earliest stages of net design, as outlined in the general framework presented earlier (Figure 3). While traditional geometric parameters remain useful for preliminary mesh and panel configurations, their applicability to modern bio-based and biodegradable materials under dynamic environmental conditions is still largely unverified. This gap represents a valuable direction for future research aimed at revising construction standards to enable the widespread deployment of sustainable and high-performance netting systems.

#### 4.1.2. Agricultural Nets: Environmental Requirements, Seasonal Functionality, and Emerging Materials

The design of agricultural nets is based on criteria that differ in part from those used for fishing nets. In this context, nets are employed for seasonal protection, mechanical support for crops, the containment of climbing plants or fruits, shading, and mulching, with the aim of reducing pesticide use, mitigating climatic impact on crops, and managing the cultivation cycle more sustainably.

Unlike marine nets, mechanical loads are less severe in agricultural applications, but greater importance is placed on the following:

- Resistance to environmental agents (UV radiation, humidity, thermal fluctuations);
- Dimensional stability during service;
- Functional lifespan limited to the crop cycle or specific seasons;
- Importantly, end-of-life behavior in open soil or composting conditions.

A central design parameter is mesh size and geometry, which must be optimized based on the agronomic function and operating context, as exemplified by the criteria below (see Table 3).

The choice of mesh has significant effects on the following:

- The specific weight of the net and material consumption;
- Breathability and moisture evaporation;
- Wind resistance and panel deformability;
- Biodegradability and composting compatibility.

Numerous innovative materials are currently being studied or are already employed, including biopolymers (PLA, PBAT, PHA), regenerated fibers (Lyocell), and natural composites. Although simulation models like those used in the marine sector are still unavailable, several agronomic field trials have provided meaningful results.

In the following, selected cases are presented that illustrate complementary strategies for sustainable agricultural net design: the first focusing on the development of biodegradable mulching nets for soil protection, and the second showcasing commercial solutions for biodegradable support nets tailored to climbing crops.

##### Case Study 1—Biodegradable Mulching Nets in PBAT/PLA Blends for Seasonal Horticulture

The widespread use of conventional polyethylene films and nets for mulching in agriculture has contributed significantly to soil contamination and microplastic accumulation, raising major environmental concerns. As noted by Kopitar et al. [56], the need for sustainable alternatives is particularly critical for short-cycle horticultural crops, where nets and films are deployed intensively but only for limited periods. Addressing this issue requires materials capable of delivering sufficient mechanical performance during the crop cycle while degrading effectively at the end of use without leaving persistent residues.

Recent research efforts by Maraveas [18] and Sharif et al. [35] have focused on the design and validation of biodegradable mulching nets based on PBAT/PLA blends. These materials were selected for their balance of mechanical strength, flexibility, and high biodegradation rates under soil conditions. The design aimed to create lightweight films or nets with a thickness optimized between 50 and 70 μm, ensuring mechanical stability for at least 90 days—long enough to support a complete crop cycle—while enabling over 80% biodegradation within 120 days in line with EN 17033 standards.

Mesh design was tailored to suppress weed growth while preserving breathability and moisture control for the soil. Although the exact mesh dimensions and filament diameters were not specified, the structures were intended to function similarly to microperforated films or light nets, rather than heavy reticulated meshes.

Validation involved a combination of laboratory mechanical testing, thermo-oxidative aging analysis, and extensive field trials. The biodegradable nets demonstrated consistent mechanical behavior throughout the service period and satisfactory degradation rates post-use, confirming their suitability as sustainable alternatives to conventional polyethylene products.

The convergence of Kopitar’s broader sustainability framework with the specific experimental work by Maraveas and Sharif et al [18,35]. underscores the growing interest in integrating biodegradable solutions into mainstream agricultural practices. This case exemplifies how material optimization, field validation, and regulatory alignment can collectively drive the transition toward more environmentally responsible cultivation methods.

##### Case Study 2—Commercial Biodegradable Climbing Plant Nets

Commercial solutions have also emerged for seasonal crop support. Intermas’ “TrellisNet Bio” [57] offers a PLA-based biodegradable netting for climbing plants like beans and peas, designed to support vegetative growth and degrade in composting conditions after use. Similar initiatives include natural jute nets marketed by The Farm Dream and True Products [58], offering 100% biodegradable support systems with 50 mm mesh openings, suitable for light climbing crops.

Such developments highlight the growing demand for compostable, eco-friendly nets that combine mechanical functionality with sustainable end-of-life disposal.

##### Additional Observations on Insect- and Bird-Protection Nets

Although fewer biodegradable options are currently available for insect- and bird-protection nets, promising solutions such as “InsectONet” (Ladybird Plant Care) [59] have appeared, offering plastic-free insect-protection alternatives based on natural fibers.

##### Concluding Remarks: Standards and Outlook

In the agricultural sector, the design of biodegradable nets is supported by specific standards such as UNI EN 17033 for soil-degradable plastics and EN 13432 for industrial composting requirements. However, codified engineering tools (e.g., FEM models, advanced simulations) are still lacking, meaning that most current solutions remain largely empirical and based on field experience.

The stages of integrated design for agricultural nets can follow a framework similar to that used for fishing nets, with adaptations to the agronomic context (see Figure 3).

The development potential is considerable but requires the following:

- Greater standardization of durability and soil degradation tests;
- The introduction of assisted design tools for lightweight mesh structures;
- Comparative LCA (life cycle assessment) and agronomic performance evaluations, initiatives that have already begun to emerge in recent research efforts.

### 4.2. Technical Standards for Marine and Agricultural Nets

#### 4.2.1. Performance and Biodegradability Standards for Marine Nets

In the fishing and aquaculture cage sectors, there is a well-established set of technical standards that define the geometric, mechanical, and, more recently, environmental characteristics of the materials used. Key standards include the following:

- ISO 1107: defines terminology and geometric specifications for nets, including mesh, yarn, knots, and finishes.
- ISO 1806: establishes test methods for determining knot breaking strength.
- NS 9415 (Norway): national standard specifying structural and performance requirements for marine aquaculture systems, including nets, in relation to environmental loads such as currents, waves, and wind.
- ASTM D6691/ISO 19679: international standards used to evaluate aerobic biodegradability of plastic materials in marine environments, also applied to nets made from compostable polymers.

These standards provide detailed methods for determining properties such as tensile strength, knot strength, deformability, and behavior under simulated environmental conditions. However, it should be noted that most of these standards were developed for conventional synthetic materials (such as PA, PE, or PP) and may be partially inadequate or not directly applicable to innovative biodegradable or bio-based materials.

Recent experimental studies have highlighted the need to adapt test conditions to the specific features of new materials. For example, Kim [1] used ISO 19679-based protocols but modified environmental conditions by incorporating sediment–water interfaces and selected marine microorganisms to realistically simulate the degradation dynamics of nets on the seafloor.

These evolving requirements are pushing regulatory frameworks to reconsider testing methodologies, particularly for emerging biodegradable and bio-based nets, which behave differently from conventional synthetic materials.

#### 4.2.2. Performance and Biodegradability Standards for Agricultural Nets

In agriculture, the regulatory framework is more fragmented and less homogeneous, with many variables depending on crop type, specific net function (shading, weather protection, containment, etc.), and geographic location. Frequently adopted standards include the following:

- ASTM D1709: measures impact resistance of plastic films and can be extended to certain lightweight nets.
- ISO 10319: defines tensile strength test methods for geotextiles, useful for analyzing mulching or containment nets.
- UNI 11325-4: Italian standard that sets minimum performance requirements for anti-hail and windbreak nets, including specifications on mechanical strength and dimensional stability.

Despite the existence of these references, many agricultural producers still rely on non-standardized internal tests, often varying by country or distributor. As observed by Briassoulis [2,3], there is a lack of shared protocols for assessing biodegradable agricultural nets, with significant heterogeneity in acceptance criteria. This regulatory gap limits the large-scale adoption of sustainable solutions and hinders product comparability across markets.

**Figure 5:**
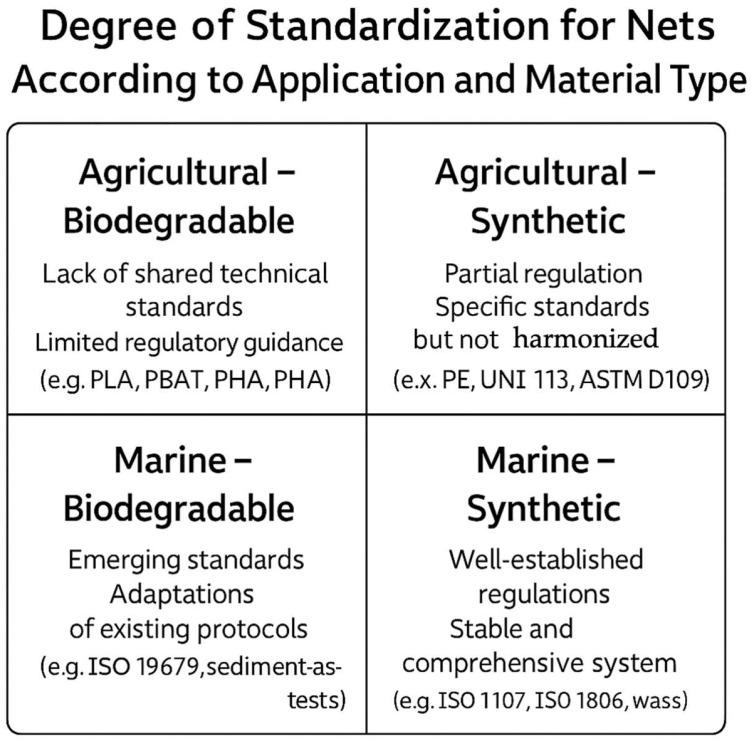
Degree of regulatory consolidation by application and material type: Overview of the current level of technical standardization for materials used in marine and agricultural nets. Synthetic nets for marine use are supported by a well-established and structured regulatory framework, whereas biodegradable materials—particularly in the agricultural sector—lack shared standards, hindering their adoption despite the growing interest in sustainable solutions.

Figure 5 graphically illustrates the degree of regulatory consolidation based on application type (marine or agricultural) and the nature of the material used (synthetic or biodegradable). It shows that synthetic nets for marine use are covered by a robust and well-defined regulatory system, resulting from decades of regulation and testing in critical areas like industrial fishing and aquaculture. In contrast, biodegradable nets for agricultural applications occupy a regulatory “gray area”, where the lack of shared technical standards hinders the dissemination of innovative materials, despite the growing demand for sustainable solutions.

### 4.3. Toward Integrated and Sustainable Design

Today, designing a net no longer means simply selecting a material or defining mesh geometry; it requires integrating traditional parameters with mechanical performance, environmental degradability, and regulatory compliance into a unified and forward-looking design strategy. This shift reflects a new paradigm, where nets are conceived not only as technical objects but as systems governed by multiple interdependent constraints.

The four key pillars of this integrated approach are as follows:

- Material and geometry, the foundational aspects of mesh design, including yarn selection, mesh size, and fabrication method;
- Mechanical performance, related to strength, elasticity, durability, and load resistance;
- Environmental degradability, the ability of materials to decompose under specific conditions without harmful residues;

Regulatory compliance, the alignment with technical and environmental standards specific to each application domain. Rather than treating these aspects separately, the integrated approach emphasizes their convergence through the following:

- The application-specific customization of lifetime, recovery strategies, and end-of-life solutions (e.g., reuse, composting, marine degradation);
- Numerical simulations validated by experimental data, enabling the prediction and optimization of structural response under real operating conditions;
- Environmental assessment tools, such as life cycle analysis (LCA) and eco-indicators, to compare material options and assess their impact across the entire life cycle.

This methodology enables the design of temporary agricultural nets optimized for lightness and biodegradability, or marine nets engineered for long-term mechanical performance and resistance to biofouling. In both cases, sustainability becomes a structural constraint—no longer an afterthought.

**Figure 6:**
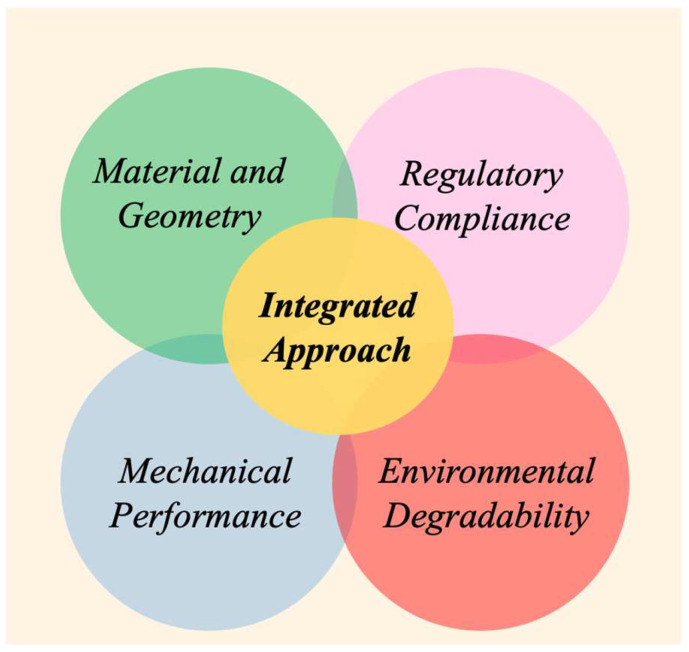
The evolving design logic for technical nets: This Venn diagram illustrates the convergence of traditional and emerging design criteria in the development of next-generation technical nets. Material and geometry—once the primary focus—are now integrated with mechanical performance, environmental degradability, and regulatory compliance. The central intersection defines the new design space where all dimensions are jointly considered, enabling truly sustainable, functional, and regulation-ready net solutions.

Figure 6 illustrates this convergence of design domains and emphasizes that only by operating at the intersection of all four areas—material and geometry, mechanical performance, environmental degradability, and regulatory compliance—can a truly integrated approach be achieved.

Yet this integration is not always straightforward. The use of biodegradable materials, for instance, does not automatically ensure equivalent performance to traditional polymers. Casanova et al. [47] highlighted this in their study comparing PBS and PA6 longline nets for tuna fishing; although both nets shared similar design, the biodegradable version showed significantly lower catch efficiency. This was attributed to physical differences such as stiffness and optical properties, underscoring that adopting new materials requires thoughtful design adaptation—not simple substitution.

The same principle applies beyond the net itself. For example, Zudaire et al. [60] tested biodegradable ropes for fish aggregating devices (FADs), aiming to reduce the long-term impact of accidental losses at sea. Their results showed that with appropriate material formulation and environmental conditioning, ropes could be engineered to gradually lose structural integrity. This demonstrates that the logic of integrated design can—and should—extend to all functional components exposed to marine environments.

## 5. Sustainable Netting Materials: Current Trends and Future Directions

In recent years, the growing focus on environmental sustainability has led the marine and agricultural netting sector to explore alternatives to conventional polymeric materials, shifting toward systems that are more environmentally compatible in both composition and life cycle management. This section analyzes the main research and development trends, highlighting solutions already applicable as well as those still under exploration.

### 5.1. Biopolymers and Biodegradable Polymer Blends

The use of biodegradable polymers represents one of the most promising strategies for reducing the environmental impact associated with the dispersion of technical nets. Materials such as polylactic acid (PLA), polyhydroxyalkanoates (PHAs), and polybutylene adipate-co-terephthalate (PBAT) have already been tested in real-world applications, both in fisheries and agriculture, demonstrating effective degradation in controlled conditions. Among these, PHA has shown particular effectiveness even in marine environments [1,6].

However, the large-scale adoption of these biodegradable materials still faces several technical and economic challenges:

- The need to improve mechanical strength, generally lower than in traditional synthetic polymers;
- The need to fine-tune degradation timing and conditions according to the application context;
- High production costs compared to conventional materials.

Recent research has focused on formulating polymer blends and copolymers capable of enhancing mechanical performance without compromising biodegradability. In particular, the integration of nanofillers—such as cellulose nanocrystals (CNCs)—has enabled the development of biodegradable mesh fibers with mechanical properties approaching those of technical polymers [7].

More advanced solutions are inspired by the structure of mussel byssus, as in the case of the fibers developed by Hong [11], composed of a PBAT core coated with a PBS shell. This core–shell configuration offers an advantageous combination of drapability, mechanical strength, and selective degradation. These fibers have also shown the ability to support controlled algae growth (e.g., *Pyropia yezoensis*), opening up interesting prospects for marine reforestation applications. In particular, Briassoulis [3] emphasized how environmental factors such as temperature, humidity, and solar radiation significantly influence the degradation rate and mode of biodegradable agricultural nets. These findings confirm the need for exposure testing under realistic conditions to accurately predict in-field performance.

**Figure 7:**
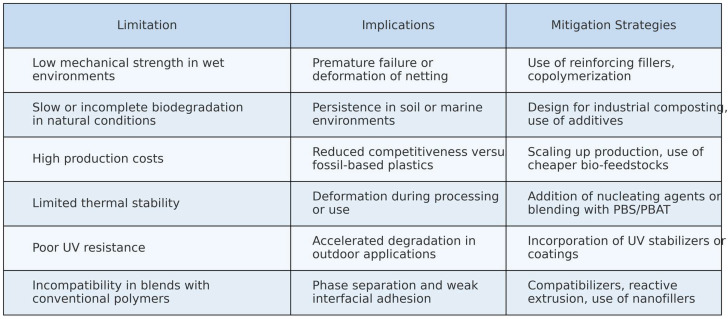
Schematic overview of key limitations associated with biodegradable polymers for netting applications and representative strategies to overcome them. The issues and solutions illustrated reflect those discussed in Section 5.1, including approaches aimed at improving mechanical strength, environmental resistance, and processing compatibility.

These considerations are summarized in Figure 7, which outlines the main limitations of biodegradable polymers in netting applications and the corresponding strategies proposed in the recent literature to address them.

### 5.2. Recycled Polymers, Hybrid Composites, and Circular Strategies

A second line of sustainable development concerns the recovery and valorization of discarded nets, particularly those made of polyamide (PA-6) from industrial fishing. These materials can undergo mechanical or chemical regeneration and be reintegrated into production cycles—not only to manufacture new nets but also to produce structural composites for construction or mid-range technical components [8].

Although this circular economy strategy does not solve the issue of non-biodegradability, it allows for the following:

- Significantly extending material service life;
- Reducing reliance on virgin raw materials;
- Supporting the development of recycling-based spinning processes for technically viable new nets.

Recent experiences have shown the possibility of combining recycled polymers, such as regenerated PA6, with bio-based matrices or coatings to obtain hybrid solutions that integrate good mechanical performance with improved environmental compatibility.

The combined use of recycled composites and natural or biodegradable fibers is the subject of emerging studies, aimed at combining the mechanical efficiency of recycled nylon with the environmental sustainability of bio-based reinforcements or matrices.

However, not all recycled materials are suitable for re-spinning into new nets. Studies on discarded polyethylene (PE) nets, such as the one by Juan et al. [61], have shown that contamination and the loss of key mechanical properties—such as crack resistance and elongation at break—make reuse for high-stress applications difficult. In such cases, alternative uses in technical components or non-food packaging can still contribute to circularity goals.

A promising area of research involves the use of structural natural fibers—such as flax, hemp, jute, or coconut—in sustainable net composites, partially or entirely replacing traditional synthetic reinforcements. These fibers, abundant, renewable, and often derived from agricultural waste, can reduce plastic content and improve composite biodegradability or controlled compostability. Preliminary results on plant fiber-reinforced biocomposites show good mechanical properties, especially when surface treatments are applied to enhance fiber–matrix adhesion [62].

However, their use in nets exposed to marine or intensive agricultural environments still requires optimization in terms of durability, water absorption, and dimensional stability. The integration of natural fibers in additive manufacturing, combined with bio-based polymers like PLA and PBAT, remains an experimental avenue. While there are studies on biocomposites and 3D printing using hydrogels or polymer blends, consolidated high-performance mesh net solutions have yet to emerge. Animal-based fibers such as feathers and bristles, though rich in keratin, show significant limitations in mechanical performance, dimensional stability, and compatibility with mesh processing technologies, making them currently unsuitable for technical nets exposed to harsh environmental conditions [63].

### 5.3. Natural Fibers, Advanced Processing, and Functional Design

The potential of sustainable composites, particularly those incorporating natural fibers, enables interesting scenarios in the field of manufacturing. The choice of production process will increasingly depend on the nature of the composite used, the need to ensure durability and specific functionality, and compatibility with emerging technologies such as the 3D printing of bio-based materials [64].

Among the most promising approaches, extrusion-based 3D printing techniques such as fused deposition modeling (FDM) and direct ink writing (DIW) have been adapted to produce hydrogel-doped or cellulose-reinforced composites, allowing variable-density mesh structures optimized for environmental conditions or localized mechanical demands [7,64].

Biodegradable fibers incorporating PBAT and cellulose nanocrystals (CNCs) have already shown mechanical properties comparable to technical polymers and potential applicability in functional net structures [7].

Recent studies also suggest the potential for marine-grade biodegradable fibers with enhanced drapability and selective degradation, such as PBS-coated PBAT core–shell fibers inspired by mussel byssus [11]. These fibers have demonstrated suitability for algae cultivation supports and are seen as candidates for multifunctional netting in restoration or aquaculture contexts.

Despite ongoing challenges in scalability and durability, the integration of natural fibers in additive manufacturing processes remains a promising path, particularly when combined with reinforcement strategies or surface modifications.

The most advanced research trends converge on the development of multifunctional nets, designed to achieve the following:

- Withstand mechanical stresses typical of marine and open-field applications;
- Degrade selectively;
- Interact with the environment (e.g., via visual degradation indicators or antibacterial properties).

Experimental developments are also exploring functionalized coatings and bioactive yarns capable of resisting biofouling and chemically interacting with their surroundings.

Some prototype materials exhibit visual responses to pH or temperature changes, while others are formulated to release antimicrobial agents in a controlled manner [63,64].

For example, recent work has demonstrated the use of functional biopolymer filaments in 3D-printed open structures designed to facilitate selective colonization by marine organisms or to deliver slow-release protective compounds [62].

Although many of these systems remain at the experimental stage, they represent a promising direction toward netting that combines structural, environmental, and sensory functionality.

In parallel, the idea of adaptive design is gaining ground—where materials and geometries are selected based on the desired lifespan, operating environment, and disposal method—integrating performance and sustainability requirements from the earliest design phases.

In this context, it will be crucial to realize the following:

- Expand and update technical standards, which are still inadequate for new generations of materials;
- Consolidate environmental assessment tools such as LCA and ecological risk analysis;
- Build production chains capable of supporting new high-performance, low-impact mesh materials.

Life cycle assessments of nets, as shown by Singh [38], demonstrate that the use of bio-based polymers and improved end-of-life management can significantly reduce overall environmental impact.

Recent solutions tested by Kim [1] confirmed the effectiveness of controlled degradation in simulated marine sediment environments, paving the way for time-programmed nets designed to disintegrate after a defined period.

**Figure 8:**
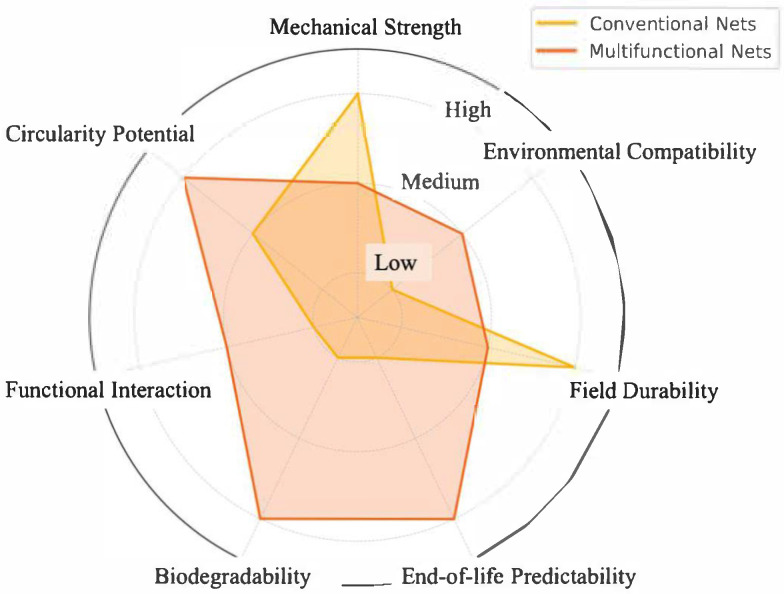
Qualitative comparison between conventional and emerging multifunctional nets: Radar chart comparing seven key design parameters: mechanical strength, environmental compatibility, in-field durability, end-of-life predictability, biodegradability, functional interaction, and circularity potential. Each parameter is qualitatively evaluated on a three-level scale (low, medium, high). The chart highlights the superior performance of multifunctional nets in sustainability-related aspects, while conventional nets retain advantages in mechanical strength and field durability.

Figure 8 presents a qualitative comparison between conventional polymeric nets and emerging multifunctional systems, evaluated across seven relevant design dimensions. While traditional materials offer established mechanical performance and high durability, multifunctional solutions show greater potential in terms of environmental compatibility, end-of-life programmability, and functional interaction with the environment. This comparison underscores the need for integrated design strategies capable of balancing structural performance with sustainability requirements.

### 5.4. International Programs and Pilot Initiatives for Biodegradable Nets

The issue of reducing the environmental impact of conventional polymer nets has become a global concern, with numerous pilot projects and research initiatives launched across all continents. These efforts aim to mitigate plastic pollution in marine ecosystems, improve the sustainability of aquaculture and agriculture, and promote the adoption of biodegradable or recyclable materials.

Although the number of initiatives is rapidly increasing, the list presented in this section is not exhaustive. It highlights a selection of representative and publicly documented projects—either completed or ongoing—classified by application area. A distinction is made between marine-related initiatives (Section 5.4.1), those targeting agricultural or forestry applications (Section 5.4.2), and comparative insights including commercial developments (Section 5.4.3), each followed by a summary table or a synthetic discussion of their main characteristics.

#### 5.4.1. Projects on Marine-Biodegradable Nets

- LIFE MUSCLES (Marine Use of Sustainable Compostable Light-Weight Eco-friendly Socks)—Replacement of conventional polypropylene socks in mussel farming with compostable biopolymers and implementation of an on-site recycling model (EU LIFE Programme; 2021–2025; Italy/European Union). Status: ongoing [65].
- Elba Island Posidonia Project—Deployment of marine-biodegradable nets to support Posidonia oceanica restoration without leaving plastic residues (local public–private collaboration; 2023; Italy). Status: ongoing [66,67].
- TEFIBIO (Test d’Engins de Pêche Biodégradables)—Development and field testing of biodegradable, bio-sourced, and recyclable trammel-type fishing nets with recycling feasibility into compost (European Maritime and Fisheries Fund—FEAMP; 2019–2023; France). Status: concluded. Result: biodegradable nets validated for compostability and functional use [68,69].
- SFI Dsolve (Sustainable Fishing Gear for Reduction of Marine Plastic Pollution)—Research on biodegradable fishing gear (nets, ropes, lines) capable of degrading naturally without releasing microplastics (Research Council of Norway—RCN; 2020–2028; Norway). Status: ongoing [70].
- Glaukos (Triggerable Bio-based Textile Polymers with Tailored Degradation and Recycling Properties)—Design of new bio-based, biodegradable, and recyclable fibers and coatings for fishing nets and textiles, integrated into a circular life cycle approach (Horizon 2020 Programme—EU; 2020–2024; European Union). Status: concluded. Results: development of bio-based and biodegradable fishing nets with marine-safe coatings, designed for controlled degradation and integration into a circular life cycle [71].
- Biodegradable Nets—South Korea—Production and promotion of fishing nets designed to fully degrade under marine conditions within three to four years (NIFS and Ministry of Oceans and Fisheries; 2020–ongoing; South Korea). Status: ongoing [72,73].
- Biodegradable Gillnets—China—National campaign for replacing conventional gillnets with PBS-based biodegradable alternatives to prevent marine plastic pollution (Ministry of Agriculture and Rural Affairs—MARA; 2021–ongoing; China). Status: ongoing [74].
- ICAR-CIFT Biodegradable Nets (Indian Council of Agricultural Research—Central Institute of Fisheries Technology)—Development and field testing of biodegradable PBS and PBAT nets for marine and inland fisheries, aligned with SDGs (Indian Council of Agricultural Research—ICAR; 2018–ongoing; India). Status: ongoing [75].
- GREENET (Green Fishing Net Project)—Research-driven development of prototype fishing nets from naturally degradable materials to minimize environmental impact (Queensland University of Technology—QUT; 2023 (prototype stage); Australia). Status: ongoing [76].
- Catchgreen (Catchgreen Project)—Field trials of Biodolomer^®^Ocean-based biodegradable nets for fishing and aquaculture in Africa (SMEP Program—UK FCDO and UNCTAD; 2022–ongoing; Kenya/South Africa). Status: ongoing [77,78,79].

#### 5.4.2. Projects on Agricultural Biodegradable Nets

- BBioNets (Boosting Bio-Based Technologies Adoption through Forest and Agriculture Networks)—Promotion of bio-based technologies and creation of regional networks to accelerate the use of biodegradable agricultural nets (Horizon Europe Programme; 2023–2026; European Union). Status: ongoing [80].
- CompostNet (Compostable Nets for Sustainable Agriculture)—Design of biodegradable insect exclusion nets suitable for household composting, combining durability and environmental friendliness (Auvergne-Rhône-Alpes Region; 2023–2026; France). Status: ongoing [81].
- BioNetAgro (Eco-Friendly Netting Initiative for Sustainable Agriculture)—Provision of eco-friendly nets to small-scale farmers for sustainable crop protection in Kenya (ICIPE—Kenya; 2017–2021; Kenya). Result: Nets successfully validated in field applications with positive outcomes in tomato farming. The BioNetAgro initiative (2017–2021), promoted by ICIPE, built upon earlier research on eco-friendly netting for tomato protection. Field validation confirmed the effectiveness of these solutions, already explored in preliminary studies (Gogo et al., 2014) [82].
- University of Queensland Bio-Packaging Project—Research on biodegradable packaging and agricultural netting solutions derived from agricultural waste (e.g., sugarcane fibers), aimed at replacing conventional plastics in farming applications (University of Queensland research funding; 2020–ongoing; Australia). Status: ongoing [83].
- MEDLIFE Greenhouse Project—Construction of family greenhouses to improve the agricultural capacity of the Chahauitiri community near Cusco, Peru, enabling vegetable cultivation despite the high-altitude climate (MEDLIFE; 2020–ongoing; Peru). Status: ongoing [84].

#### 5.4.3. Comparative Insights and Commercial Developments

A comparative analysis of the two sectors provides useful insights into the current landscape of biodegradable netting systems. While marine-related applications appear to be more extensively supported by structured programs, agricultural initiatives are emerging more recently and with fewer examples.

A comparison between Table 4 and Table 5 reveals this imbalance clearly: the marine sector has seen the implementation of numerous projects—often backed by European, national, or academic frameworks—whereas the agricultural field remains under-represented in structured efforts. This discrepancy suggests a temporal gap in both the recognition of the problem and the organized response to the environmental impact of agricultural netting systems.

Moreover, the vast majority of the initiatives listed are both recent and still ongoing, indicating a rapidly growing international interest in replacing conventional polymer-based nets with biodegradable alternatives. This temporal clustering reflects the mounting urgency surrounding plastic pollution and aligns with broader global sustainability objectives, particularly those related to climate neutrality, biodiversity protection, and circular economy principles.

In parallel with public and research-driven initiatives, several private companies have launched commercial products based on starch or other bio-based polymers. These solutions are mainly intended for forestry, viticulture, horticulture, and turfgrass management. Notable examples include the following:

- The NetPlus^®^ Program (Bureo; 2013–ongoing), which recovers and recycles discarded fishing nets into consumer goods within a circular materials model [85];
- The Nets for Change initiative (NBA and World Surf League; 2024–ongoing), which upcycles ghost nets into basketball nets for use in community courts [86];
- The BioPBS Turf Net Project (PTT MCC Biochem; 2020–2023), which demonstrated the technical viability of biodegradable turfgrass netting [87];
- The PROTECTNET BIO and CLIMATIC BIO product lines from Intermas (2022–ongoing), developed for biodegradable crop and plant protection [88] and additional commercial solutions from InCord and Dylaa Handicrafts [89,90], currently marketed for horticultural and agricultural applications.

These industrial initiatives—though not structured as formal research projects—complement experimental and pilot-scale programs by accelerating the availability of biodegradable netting solutions on the market. Together, these public and private efforts illustrate a widespread and increasing interest in sustainable netting systems. The integration of biodegradable polymers, compostability, in-field durability, and recyclability represents a central axis of innovation. Embedding these advancements into technical guidelines and regulatory frameworks will be essential for transitioning from demonstrative applications to systemic change in both marine and terrestrial sectors.

## 6. Discussion

The growing focus on environmental sustainability and the need to reduce the impact of dispersed or improperly disposed nets have led to significant advancements in the material and design criteria for nets used in marine and agricultural applications. This work has provided an overview of polymeric and composite materials used in net manufacturing, offering an in-depth analysis encompassing structural, functional, environmental, and regulatory aspects.

While conventional nets made from synthetic polymers are mechanically efficient, they pose major challenges in terms of environmental degradation and end-of-life management. In response, innovative solutions have emerged based on biodegradable polymers (such as PLA, PHA, and PBAT), optimized blends, and nanocomposites, as well as regenerated materials from discarded nets. These approaches represent a concrete step toward the integration of sustainability and functionality, though they still require regulatory and application-level consolidation.

In parallel, design and experimental activities at both European and international levels have produced numerous examples of biodegradable nets applied in fisheries, aquaculture, agriculture, and environmental restoration. These experiences demonstrate the technical feasibility of such solutions and provide valuable data for future standardization, while also highlighting the need for shared protocols for environmental and performance testing.

Overall, the future of technical nets lies in the ability to combine reliable mechanical performance with ecological compatibility, through the adoption of multifunctional materials, adaptive design, and circular strategies. An integrated approach must begin at the design stage, incorporating realistic experimental data and LCA tools to ensure not only environmental impact reduction but also life cycle efficiency.

Looking ahead, several development paths appear particularly promising. Notably, the design of nets based on reinforced biodegradable blends—such as PBAT-based matrices with nanofillers (e.g., CNC) or core–shell structures inspired by mussel byssus—offers a concrete path toward durable and programmable mesh systems, as demonstrated in recent studies [6,12]. However, the challenge remains in ensuring effective degradation under real conditions, especially in sediment–water interaction zones, as pointed out by Kim [1]. In this regard, more targeted and adaptable environmental testing protocols must be developed based on usage context.

In the field of regenerated materials, the combination of recycled synthetic fibers with bio-based matrices opens up new possibilities for high-performance hybrid solutions. The technical potential has been confirmed, but process standardization and performance requirements for such secondary materials are still lacking. Moreover, recycling supply chains must be reorganized in a more functional and decentralized manner to enable local valorization [7].

One still-underexplored area is that of multifunctional nets—devices capable not only of mechanical action but also of serving as biological substrates, environmental sensors, or temporary supports for marine or agricultural ecosystems. The example of ABf fibers developed for marine afforestation suggests that nets can be reimagined not merely as technical objects but as active interfaces between natural systems and infrastructure.

Finally, integrating these innovations into the regulatory framework requires a dual approach: on the one hand, updating existing standards to accommodate new materials and testing methods; and on the other, defining reference parameters for mechanical performance, functional lifespan, and context-specific biodegradability (marine or agricultural, permanent or seasonal use).

Only a joint approach—combining applied research, ecosystem-based design, regulatory validation, and industrial development—can enable the structural adoption of sustainable nets, ensuring not only technological innovation but also its scalability and large-scale implementation.

Moreover, the comparative analysis between marine and agricultural applications highlights the importance of cross-sectoral learning. Experiences gained in the marine sector, where structured projects have been more numerous, could accelerate the adoption and optimization of biodegradable nets in agriculture. Bridging this gap through the harmonization of design, testing, and regulatory approaches will be crucial to enable the large-scale deployment of sustainable netting systems across different environmental contexts.

Beyond material and structural innovations, the true shift lies in redefining nets as active tools for ecological integration. This requires not only advanced design and testing, but also the institutional and industrial will to scale sustainable solutions into global practice.

## 7. Conclusions

The development of sustainable netting systems demands a paradigm shift—one that combines advanced materials, integrated design methodologies, and context-specific environmental validation. The studies and initiatives reviewed in this work confirm the feasibility of this transition. Moving from experimental solutions to scalable implementations will depend on harmonized standards, robust field testing, and collaborative frameworks between research, industry, and regulatory bodies.

## Figures and Tables

**Table 1:** (**A**) Main net configurations. (**B**) Less common or emerging configurations.

Configuration	Application Field	Materials	Notes	References
(**A**)				
Knotted net	Marine	PA, PE	High stability, visible knots, abrasion resistance	[10,12]
Knotless net	Marine, Agricultural	PA, PE, PLA	Flexible mesh, low fish damage, knot-free	[2,10]
Raschel net	Marine (limited), Agricultural	HDPE, PP, PLA	Chain-like mesh, flexible, suitable for seasonal use	[2,13]
Leno weave	Agricultural	HDPE, PP	Rigid interlacing, good dimensional stability	[10,13]
Flat woven net	Marine (limited), Agricultural	HDPE, PA	Regular mesh, moderate structural resistance	[10]
Braided rope	Marine, Agricultural (structural)	PE, PA, twisted synthetic fibers	High tenacity, used for edges and structural support	[5]
(**B**)				
Hexagonal mesh	Marine (limited)	PA, HDPE	Flexible hexagonal openings, used for mollusk containment	[5,9]
Spiral netting	Aquaculture (modular nets)	Metal wires	Helical structure with lateral walls interconnected through spirals, enhancing flexibility and mechanical strength	[14]
Strap net	Agricultural (mulching)	Biodegradable PE	Perforated band-like mesh, ground stability	[2,3]
Thermofused net	Marine (evolved knotless)	PA	Heat-welded mesh, regular shape and knotless	[9]
3D multilayer net	Experimental	Bio-based composites	Multilayer net structures for advanced aquaculture cages	[7,8]

**Table 2:** Mechanical properties of selected fossil-based, bio-based, recycled, and composite polymers used in netting systems for marine and agricultural applications. Values are reported as ranges from data in the literature and reflect typical formulations used or proposed in the context of tensile elements or net filaments.

Material	Fiber Diameter (μm)	Tensile Strength (MPa)	Elastic Modulus (GPa)	Elongation at Break (%)	Reference(s)
**PA6 (nylon)**	10–50	700–1000	3.9–6.0	10–15	[24]
**HDPE**	300	403–441	1	27–31	[25]
**PP**	10–150	200–700	0.5–9.8	10–15	[24]
**PE**	40	400	2–4	100–400	[24]
**Recycled PA6**	bulk	47	2.6	12	[26]
**PA6/15% biochar composite**	bulk	47	4.5	6.3	[26]
**PLA**	10–50	74	3.1	3.3	[27]
**PBAT**	film	17.7	0.0016	1207.6	[28]
**PBS**	bulk	34	0.254	23.1	[29]
**Mater-Bi^®^**	“	11.7	0.18	95	[30]
**PLA/flax composite**	“	35–66	3–5	-	[31,32]
**PP/flax composite**	“	45–57	6.5	-	[33,34]

**Table 3:** Criteria for agricultural nets based on agronomic function.

Agronomic Function	Mesh Design Criteria
Insect protection	Mesh size < 2 mm (physical barrier against small insects)
Bird protection	Mesh size 15–20 mm (corresponding to target bird body size)
Support for climbing plants	Mesh size 30–50 mm (compatibility with stems and tendrils)
Shading	Dense weaving, coverage ≥ 50%, oriented for solar shading
Mulching	Dense mesh or microperforated film, thickness < 100 μm

**Table 4:** International projects on biodegradable nets for marine applications. Selection of public and academic initiatives focused on the development and testing of biodegradable nets for marine applications, including mussel farming, commercial fishing, environmental restoration, and aquaculture. The technologies involved range from compostable biopolymers to bio-based materials designed to degrade in seawater. Projects have been implemented across Europe, Asia, Africa, Australia, and Latin America.

Project/Initiative	Application Area	Materials/Technologies	Country/Coordination	Period
LIFE MUSCLES	Mussel farming (tubular nets)	Compostable biopolymers, mechanical recycling	Italy/EU	2021–2025
Elba Island Posidonia	Seagrass restoration	Marine-biodegradable bioplastic yarns	Italy	2023–2026
TEFIBIO Project	Fishing nets (trammel type)	Biodegradable, bio-sourced materials	France	2019–2023
Dsolve Project	Fishing gear	Biodegradable synthetic replacements	Norway	2020–2028
Glaukos Project	Fishing nets and textiles	Bio-based, biodegradable fibers and coatings	European Union	2020–2024
Biodegradable Nets	Fishing nets	Biodegradable materials (ANKOR Bioplastics)	South Korea	2020–ongoing
Biodegradable Gillnets	Gillnets	PBS biodegradable polymers	China	2021–ongoing
ICAR-CIFT Biodegradable Nets	Marine and inland fishing nets	PBS and PBAT polymers	India	2018–ongoing
GREENET Project	Biodegradable nets	Naturally degradable materials	Australia	2023 (prototype)
Catchgreen Project	Marine and freshwater fishing nets	Marine-degradable Biodolomer®Ocean (PBS/PBAT)	Kenya/South Africa	2022–ongoing

**Table 5:** International projects on biodegradable nets for agricultural applications. Overview of structured projects funded by public programs or led by research institutions, aimed at promoting biodegradable nets in the agricultural sector. Applications include crop protection, insect exclusion, family greenhouses, and the development of compostable materials from plant biomass. The projects span Europe, Africa, Oceania, and South America.

Project/Initiative	Application Area	Materials/Technologies	Country/Coordination	Period
BBioNets	Agriculture/Forestry	Bio-based materials from plant biomass	European Union	2023–2026
CompostNet	Insect exclusion nets	Compostable biomaterials	France (Auvergne-Rhône-Alpes)	2023–2026
BioNetAgro	Crop protection (tomato farming)	Eco-friendly agricultural netting	Kenya (ICIPE)	2017–2021
University of Queensland Bio-Packaging Project	Agricultural packaging and netting	Biodegradable composites from agricultural waste	Australia (University of Queensland)	2023–ongoing
MEDLIFE Greenhouse Project	High-altitude community agriculture	Greenhouse nets and structures	Peru (MEDLIFE)	2020–ongoing

## Data Availability

No new data were created or analyzed in this study. Data sharing is not applicable to this article.

## Abbreviations

The following abbreviations are used in this manuscript:

PLA	Polylactic Acid
PHA	Polyhydroxyalkanoates
PBAT	Polybutylene Adipate-co-Terephthalate
PBS	Polybutylene Succinate
CNCs	Cellulose Nanocrystals
FEM	Finite Element Modeling
LCA	Life Cycle Assessment
SUP	Single-Use Plastics
EMFAF	European Maritime, Fisheries and Aquaculture Fund
